# Optimized sample preparation and data analysis for TMT proteomic analysis of cerebrospinal fluid applied to the identification of Alzheimer’s disease biomarkers

**DOI:** 10.1186/s12014-022-09354-0

**Published:** 2022-05-14

**Authors:** Sophia Weiner, Mathias Sauer, Pieter Jelle Visser, Betty M. Tijms, Egor Vorontsov, Kaj Blennow, Henrik Zetterberg, Johan Gobom

**Affiliations:** 1 Department of Psychiatry and Neurochemistry, Institute of Neuroscience and Physiology, University of Gothenburg, Mölndal, Sweden; 2 Clinical Neurochemistry Lab, Institute of Neuroscience and Physiology, Sahlgrenska University Hospital, Mölndal, Sweden; 3 Department of Psychiatry and Neuropsychology, Alzheimer Centrum Limburg, School for Mental Health and Neuroscience, Maastricht University, Maastricht, The Netherlands; 4 Present Address: Department of Neurology, Alzheimer Center Amsterdam, Amsterdam Neurosciences, Vrije Universiteit Amsterdam, Amsterdam UMC, Amsterdam, The Netherlands; 5 Division of Neurogeriatrics, Department of Neurobiology, Care Sciences and Society, Karolinska Institutet, Stockholm, Sweden; 6 Proteomics Core Facility, Sahlgrenska Academy, University of Gothenburg, Gothenburg, Sweden; 7 Department of Neurodegenerative Disease, UCL Institute of Neurology, London, UK; 8 UK Dementia Research Institute, London, UK; 9 Hong Kong Center for Neurodegenerative Diseases, Hong Kong, China

**Keywords:** Tandem mass tag, Cerebrospinal fluid, Normalization, Alzheimer’s disease, Biomarkers, Sample preparation, Labeling efficiency, Mass spectrometry

## Abstract

**Background:**

Cerebrospinal fluid (CSF) is an important biofluid for biomarkers of neurodegenerative diseases such as Alzheimer’s disease (AD). By employing tandem mass tag (TMT) proteomics, thousands of proteins can be quantified simultaneously in large cohorts, making it a powerful tool for biomarker discovery. However, TMT proteomics in CSF is associated with analytical challenges regarding sample preparation and data processing. In this study we address those challenges ranging from data normalization over sample preparation to sample analysis.

**Method:**

Using liquid chromatography coupled to mass-spectrometry (LC–MS), we analyzed TMT multiplex samples consisting of either identical or individual CSF samples, evaluated quantification accuracy and tested the performance of different data normalization approaches. We examined MS2 and MS3 acquisition strategies regarding accuracy of quantification and performed a comparative evaluation of filter-assisted sample preparation (FASP) and an in-solution protocol. Finally, four normalization approaches (median, quantile, Total Peptide Amount, TAMPOR) were applied to the previously published European Medical Information Framework Alzheimer’s Disease Multimodal Biomarker Discovery (EMIF-AD MBD) dataset.

**Results:**

The correlation of measured TMT reporter ratios with spiked-in standard peptide amounts was significantly lower for TMT multiplexes composed of individual CSF samples compared with those composed of aliquots of a single CSF pool, demonstrating that the heterogeneous CSF sample composition influences TMT quantitation. Comparison of TMT reporter normalization methods showed that the correlation could be improved by applying median- and quantile-based normalization. The slope was improved by acquiring data in MS3 mode, albeit at the expense of a 29% decrease in the number of identified proteins. FASP and in-solution sample preparation of CSF samples showed a 73% overlap in identified proteins. Finally, using optimized data normalization, we present a list of 64 biomarker candidates (clinical AD vs. controls, p < 0.01) identified in the EMIF-AD cohort.

**Conclusion:**

We have evaluated several analytical aspects of TMT proteomics in CSF. The results of our study provide practical guidelines to improve the accuracy of quantification and aid in the design of sample preparation and analytical protocol. The AD biomarker list extracted from the EMIF-AD cohort can provide a valuable basis for future biomarker studies and help elucidate pathogenic mechanisms in AD.

**Supplementary Information:**

The online version contains supplementary material available at 10.1186/s12014-022-09354-0.

## Background

Cerebrospinal fluid (CSF) is the primary biofluid for biomarkers of central nervous system diseases. Due to its proximity to the brain, many neuropathological processes are mirrored in the protein composition of the CSF [1]. In Alzheimer’s disease (AD) research, the CSF biomarkers amyloid β (a marker of amyloid plaque pathology), and the tau protein (a marker of axonal destabilization) with its phosphorylated forms (markers of increased tau phosphorylation, which are related to neurofibrillary tangle formation) have played central roles for our current understanding of the pathological mechanisms involved in the disease [2]. They have aided drug development and are gaining importance as a support in diagnostics and as indicators of disease progression [3]. Identification of more CSF biomarkers through mass spectrometry (MS)-based proteomics is likely to facilitate the transition from symptomology-based definitions of neurodegenerative diseases to a more precise one defined by specific pathologies that can be determined by measuring panels of biomarkers. This may assist the development of treatments, help identify disease subtypes, and ensure that patients receive the medication suitable to treat their specific pathologies.

Precise protein quantitation improves the ability to identify biomarkers. The tandem mass tag (TMT) technique is one of the most frequently used techniques for quantifying relative protein abundances in proteomic studies [4]. In a TMT experiment, protein samples subjected to tryptic digestion are labeled with an amino-reactive reagent. TMT reagents exist in different isobaric forms, each differing in how the stable isotopes ^13^C and ^15^N are distributed between the reporter and balance group of the reagent [5]. TMT-labeled study samples are combined into multiplexes that are analyzed by liquid chromatography mass-spectrometry (LC–MS) in the data-dependent mode using high-resolution MS. Upon fragmentation of the precursor, the reporter part of the label is released and detected in the fragment ion spectrum. As reporter ions from different TMT reagents have distinct *m/z* values, they are detected as separate peaks, the relative intensities of which correspond to the relative molar concentration of the fragmented peptide ion across the study samples contained in the TMT multiplex sample [6]. To date, the TMTpro technique enables multiplexing of up to 18 samples that can be analyzed simultaneously in one LC–MS run [7]. Both the high multiplexing capacity and the quantitation accuracy make TMT a powerful tool in large clinical proteomic studies [8].

However, there are also challenges associated with TMT studies in CSF. One is the large biological variability of CSF compared to e.g., cultured cells or tissue extracts [9]. The total protein concentration in CSF varies widely among individuals, ranging between 0.15 and 0.6 mg/mL [10, 11] as a result of a multitude of factors including CSF production and clearance rates, blood–brain barrier permeability and venous pressure [12]. Proteomic studies in CSF have also revealed considerable variation in the concentrations of numerous proteins between individuals [13, 14]. This raises concerns that differences in the overall protein composition may affect sample preparation yield, thereby distorting the measurement of individual proteins.

Sample preparation, including protein solubilisation, tryptic digestion, and TMT labeling may influence the results strongly, with respect both to identified proteins and to quantitation variability. If TMT labeling is performed in neat CSF, matrix effects caused by sample components may potentially interfere with the labeling reaction in a sample-dependent manner. Such matrix effects can be largely avoided using filter-assisted sample preparation (FASP) [15]. While also permitting the use of strong protein solubilizing agents, which may allow identification of proteins not accessible to tryptic digestion in solution, FASP comes with the drawback of being more laborious and time-consuming, which hampers its use in large-scale studies.

It is important to achieve near complete labeling of peptides, as incomplete labeling leads to decreased detection sensitivity. Furthermore, under reaction conditions with TMT reagent deficiency, the sample composition may conceivably affect the degree of labeling, thereby skewing measurements. On the other hand, in large-scale studies it is desirable to minimize the amount of the costly TMT reagent. Cost-efficient TMT labeling has been reported for purified protein samples and may possibly also be achievable in CSF [16].

Quantitation accuracy of TMT suffers from a phenomenon referred to as ratio compression: distinct TMT labeled peptide ions with close precursor *m/z* values are co-isolated and co-fragmented in MS2, skewing reporter ion intensities towards a 1:1 ratio [17]. To circumvent this, a method known as synchronous precursor selection (SPS) MultiNotch MS3 was developed in which multiple carefully selected MS2 fragment ions, containing the TMT label, are subjected to an additional fragmentation step [18]. While improving ratio distortion, this method comes at the expense of detection sensitivity [18]. In CSF, in which potential biomarkers are likely to be low-abundant, the gain in accuracy must be critically weighed against the decrease in detection sensitivity.

Another important aspect in TMT studies is data normalization of the obtained protein abundance values. Technical variation in the yield of sample preparation steps prior to TMT labeling, and in the labeling reaction itself, can introduce systematic differences in protein amount and composition between samples. Furthermore, high biological variation among CSF samples may obscure relevant protein changes. To correct for differential influences on the samples and achieve higher comparability, some form of normalization is typically applied to the data. Most data normalization techniques assume that the overall protein composition is similar across study samples. In the light of the high biological variability of CSF, this assumption may not hold true for CSF demanding a careful evaluation of different normalization approaches.

In this study we set out to systematically address the challenges associated with TMT studies in CSF. FASP was compared with a protocol based on in-solution tryptic digestion and TMT labeling of neat CSF, with respect to identified proteins and variability of quantification. For the latter protocol, we also determined the minimal required amount of TMT reagent. Using both pooled CSF and individual CSF samples, we evaluated the effect of previously reported normalization methods, including normalization to spiked-in external protein, Total Peptide Amount normalization, median normalization, and quantile normalization. Finally, we applied the normalization found to be optimal, to a recently published data set from the European Medical Information Framework (EMIF)-AD cohort, resulting in the identification of AD biomarker candidates [19].

## Materials and methods

### Materials

Human CSF individual (15 in total) as well as pool samples were obtained from the Neurochemistry laboratory at Sahlgrenska University hospital, Mölndal. AD core biomarkers were measured using a chemiluminescent enzyme-immunoassay (CLEIA) on the LUMIPULSE® G1200 platform (Fujirebio Europe). Samples were biochemically classified as AD based on the following cut-off values: Aβ_1–42_ < 620 pg/mL, phospho(p)-tau 181 > 61 pg/mL and total(t)-tau > 440 pg/mL.

Internal calibrator (QCAL) was prepared by dissolving 25 µg vials (Sigma-Aldrich) in 480 µL 20% acetonitrile (ACN). Aliquots of 10 µL were lyophilized with a SpeedVac vacuum concentrator yielding a peptide amount of 10 pmol/aliquot.

Reference peptide mix (MassPrep Digestion Standard Mixture 1, Waters) consists of the following proteins: yeast alcohol dehydrogenase (ADH, SwissProt P00330), glycogen phosphorylase b (GPb, SwissProt P00489), yeast enolase (SwissProt P00924), bovine serum albumin (BSA, SwissProt P02769) in molar ratios 1:0.92:0.36:0.62 (± 5–10%). Reference peptide mix (pepmix) vials were dissolved in 1 mL 0.1% formic acid (FA) and vortexed for 1 h at RT. Aliquots of 100 µL were lyophilized with a SpeedVac vacuum concentrator (final amount: 5 pmol), and reconstituted in 2.5 mL 0.1% trifluoroacetic acid (TFA), 50 µL aliquots were made (100 fmol) and stored at − 20 °C.

### In-solution CSF sample preparation

In-solution digestion was performed as previously described [20]. Briefly, 50 µL CSF aliquots were spiked with QCAL internal calibrator (50 fmol) and pepmix (0, 10, 15, 30 fmol). Samples were reduced by adding 19.5 µL 24.2 mM Tris(2-carboxyethyl)phosphine solution and heated at 55 °C for 1 h. For alkylation, 2.4 µL freshly prepared 400 mM iodoacetamide (IAA) were added to each sample followed by vortexing and a 30 min incubation in the dark. Trypsin (20 µg per vial; Promega) was dissolved in 100 µL of resuspension buffer (Promega), and an aliquot containing 2.6 µg of the enzyme was added to each sample. Samples were digested overnight at 37 °C.

### Filter-assisted sample preparation (FASP) of CSF

First, 50 µL CSF aliquots were spiked with 25 µL pepmix in 50 mM of triethylammonium bicarbonate (TEAB); 8 µL of sodium dodecyl sulfate (SDS) were added to each sample to reach a final concentration of 2%. Samples were vortexed and subsequently heated at 90 °C for 5 min. Protein reduction was performed by adding 4.4 µL dithiothreitol (DTT) to each sample and incubating at 60 °C for 30 min. The following centrifugation steps were performed at 12,000×*g* for 15 min. Filter units (30 kDa, PALL Life Science) were equilibrated by applying 200 µL 8 M Urea with subsequent centrifugation. Each sample was diluted 1:4 with 8 M Urea and added to the filter unit. Samples were spun down and SDS was removed by washing 3 times with 200 µL 8 M Urea. Having washed the filter unit with digestion buffer (0.5% sodium deoxycholate (SDC), 50 mM TEAB), samples were alkylated by adding 100 µL 18 mM IAA in digestion buffer, vortexed and incubated for 20 min in the dark. Following the removal of alkylation buffer and filter equilibration, trypsin dissolved in digestion buffer was added at enzyme:protein ration 1:60 (w/w). Samples were digested overnight at 37 °C and spun down the following day for peptide collection.

### TMT labeling and peptide desalting

Prior to labeling, sample pH was verified to be around 8. TMTpro 16plex reagents were equilibrated to room temperature and dissolved in 20 µL 100% ACN. Vials were thoroughly vortexed, spun down and the corresponding TMT volume was transferred to the sample (100% TMT volume: 20 µL, 50% TMT volume: 10 µL, 20% TMT volume: 4 µL). Samples were then incubated for 1 h with constant agitation (200 rpm). The labeling reaction was quenched by adding 5 µL of 5% hydroxylamine (HA) to the sample and incubating for 30 min. Samples were pooled into corresponding TMT sets and diluted with 0.1% TFA to decrease the ACN concentration to < 3%. One tenth of the sample volume of 0.5 M HCl was then added to acidify the samples. Desalting was performed by solid phase extraction (SPE) employing reversed-phase C_18_ cartridges (Sep-Pak C18 light) with a vacuum manifold. Cartridges were first washed with 1000 µL 0.1% TFA, 80% ACN and then equilibrated by applying 1000 µL 0.1% TFA twice. TMT sample sets were loaded onto the column and the cartridge was again washed twice with 1000 µL 0.1% TFA. Peptides were finally eluted with 1000 µL 0.1% TFA, split into 2–4 aliquots and lyophilized employing a SpeedVac vacuum concentrator.

### Offline high-pH reverse phase HPLC sample fractionation

Offline high-pH HPLC sample fractionation was performed using an UltiMate™ 3000 Nano LC system. One sample aliquot was reconstituted in 22 µL 2.5 mM NH_4_OH and 20 µL were loaded onto an XBridge BEH C18 column (pore size: 130 Å, inner diameter: 4.6 mm) for separation. Peptides were eluted over a 65 min gradient at a flow rate of 100 µL/min with Buffer B ranging from 1 to 45% and Buffer C = 10% (Buffer A: H_2_O, Buffer B: 84% ACN, Buffer C: 25 mM NH_4_OH). Fractions were collected with 1.0 min interval and concatenated to 24 fractions by circling over two rows in a 96-well microtiter plate. The column was consequently cleaned at 90% B, 10% C for 10 min and equilibrated at 1% B, 10% C for 10 min. Eluates were lyophilized by vacuum centrifugation and stored at − 20 °C until LC–MS analysis.

### Liquid chromatography–mass spectrometry (LC–MS)

Samples were analysed with a nano-LC (Ultimate RSLC Nano, Thermo Scientific) equipped with a C18 trap column (PepMap Acclaim 300 µm mm * 5 mm, Thermo Scientific), and a C18 separation column (PepMap Acclaim 75 µm * 500 mm, Thermo Scientific), coupled to a Fusion Tribrid Orbitrap mass spectrometer (Thermo Scientific), fitted with an Easy Spray ion source. Loading buffer was 0.05% TFA; Buffer A was 0.1% FA; and Buffer B was 84% ACN, 0.1% FA. The following gradient was used: 0 min, B 0%; 50 min, B 55%; 60 min, B 100%. The mass spectrometer was operated in the positive ion mode. A full Orbitrap MS scan (R = 120 k, AGC target = Standard, max injection time = 50 ms) was followed by data dependent Orbitrap MS/MS scans (isolation window = 1.5, activation type = HCD, R = 50 k, AGC target = 300%, max. injection time = 90 ms) with 3 s cycle time. For SPS-MS3, the following parameters were used: a full Orbitrap MS scan (R = 120 k, AGC target = standard, max injection time = 50 ms) was acquired, followed by Ion Trap MS/MS scans (isolation window = 0.7, activation type = CID, AGC target = Standard, max. injection time = 50 ms) and Orbitrap MS3 scans (MS2 isolation window (m/z) = 2, number of SPS precursors: 5, Activation type = HCD, AGC target = 200%, max. injection time = 105 ms).

### Data processing and protein quantification

Data analysis was performed with Proteome Discoverer Version 2.5.0.400 (Thermo Scientific). Reporter ion integration was carried out with 20 ppm tolerance and most confident centroid was set as integration method. Peptides were identified using Sequest™ search engine with UniProtKB Swiss-Prot (TaxID = 9606, *Homo sapiens*) as database. The following search settings were applied: precursor Δm tolerance = 10 ppm, fragment Δm tolerance = 0.02 Da (MS2 mode), 0.6 Da (MS3 mode), missed cleavages = 2, fixed modifications = carbamidomethyl, TMTpro (peptide N-terminus, K residues). For peptide scoring, Percolator was employed with an identification threshold of 1% false discovery rate (FDR). Peptide to protein summarization was performed as implemented by Proteome Discoverer [21], adapted from McAlister et al*.* [18]. Protein abundances are therein calculated as simple summation of their associated peptide group abundances. Peptide groups were considered for quantification based on their uniqueness (unique peptides) and in accordance with the principle of parsimony (razor peptides). No imputation of missing values was performed. For evaluation of TMT labeling efficiency, TMTpro was set as variable modification (peptide N-terminus, K residues).

### Data normalization methods

As multiple formulas will be presented in the following, notations are introduced at this point:

- **x**_**ij**_ denotes the peptide abundance of peptide j in sample i while **x̃**_**ij**_ indicates the corresponding normalized peptide abundance.
- **X**_**ij**_ is the protein abundance ratio of protein j in sample i and **X̃**_**ij**_ represents the corresponding normalized protein abundance ratio. Protein ratios were calculated based on median protein intensity across all TMT channels in the case of the individual and pool CSF data set. In the case of the EMIF-AD cohort, protein ratios were calculated based on the corresponding global internal standard (GIS) channel.
- **N** corresponds to the maximum number of peptides or proteins in sample i.
- **M** indicates the maximum number of samples within a TMT set.

#### Total peptide amount normalization

Normalization to total peptide amount aims at equalizing peptide abundances across all TMT channels and correcting for differences in sample loading. In the present study, normalization to total peptide amount was performed as implemented in Proteome Discoverer according to the following formula:

$${\tilde{\text{x}}}_{ij} = \frac{{x_{ij} *\mathop {\max }\limits_{1 \le i \ge M} \left( {\sum\limits_{j = 1}^{N} {x_{ij} } } \right)}}{{\sum\limits_{j = 1}^{N} {x_{ij} } }}.$$

#### External spike-in normalization

External spike-in normalization is targeted at correcting for unwanted technical variation introduced throughout the experiment. Therefore, an external reference standard is spiked into each sample at a known concentration. Here, QCAL peptide mix (Sigma Aldrich), designed as universal MS standard, was employed as reference. The normalization was performed in Proteome Discoverer. Reference protein abundance is calculated for each sample determining the maximum abundance in all samples. The normalization factor results as the factor of the maximum reference protein abundance of all samples and the individual abundance of the corresponding sample:

$$\tilde{X}_{ij} = \frac{{X_{ij} *\mathop {\max }\limits_{1 \le i \ge M} \left( {X_{i,QCAL} } \right)}}{{X_{i,QCAL} }},$$

where X_i,QCAL_ denotes the QCAL protein abundance in sample i.

#### Median normalization

Median normalization is a global normalization method correcting for differential sample amounts in a robust manner. It centers the sample data to its corresponding median. First, median protein abundance ratio is calculated for each sample. Next, each individual protein abundance in a channel is divided by its corresponding median.

$$\tilde{X}_{ij} = \frac{{X_{ij} }}{{{\text{median}}(X_{i} )}}.$$

#### Quantile normalization

Quantile normalization relies on the assumption that the global statistical distribution of protein abundances is similar across all samples. Following quantile normalization, the quantiles of protein ratio distribution in each sample are adjusted to the average quantile values obtained over all samples [22]. The function normalize.quantiles(), originally implemented for microarray data, was used in R [23]. The algorithm consists of the following steps:

1. Sort each column (TMT channel) according to their corresponding protein abundances.
2. Calculate the means across rows (protein observation) of the sorted data array. Assign that mean to each corresponding row element.
3. Rearrange each column to have the original ordering.

#### TAMPOR

TAMPOR is a function originally implemented by Dammer and colleagues [24]. It employs a median polish algorithm based on Tukey’s median polish correcting for both abundance differences in samples and TMT batch effects. In addition to performing sample (TMT channel)-wise median centering it also performs row (protein)-wise median centering by calculating the grand median of GIS channels in a TMT study [25].

### Statistical analysis

Statistical analysis of the EMIF-AD dataset comparing controls versus clinical AD was performed in R version R.4.1.2. For calculation of protein fold-change abundances, data were log2-transformed following normalization to satisfy the requirement of a normal distribution. Outliers of within-protein measurements were removed if they deviated more than 1.5 times the interquartile range of the 25th and 75th percentile, respectively. Unpaired Student’s t-test assuming unequal variance was performed to calculate significance levels and the resulting p-values were corrected by the method Benjamini and Hochberg (BH) [26]. An FDR of 1% was selected as cut-off.

### Assessment of labeling efficiency

Labeling efficiency was calculated as follows [27]:

$$LE\left[ \% \right] = \frac{{\sum {peptide\;group\;IDs} - \sum {peptide\;group\;IDs\;without\;TMT\;label} }}{{\sum {peptide\;group\;IDs} }}*100.$$

### Gene ontology analysis

For gene ontology (GO) analysis, the online tool PANTHER v.14.0 was employed [28, 29]. A statistical overrepresentation test of GO biological process terms was performed to evaluate whether genes mapping to our EMIF-AD biomarker candidate list were either over- or underrepresented [30]. As reference list, all identified proteins of the proteomic study of the EMIF-AD cohort was used. When conducting a statistical overrepresentation test for significantly increased proteins in AD, BH correction was performed (FDR < 5%). Due to the small number of significantly decreased proteins in the biomarker candidate list, no BH-correction was performed for the corresponding p-values.

## Results

### Evaluating variance within a TMT experiment

**Fig. 1:**
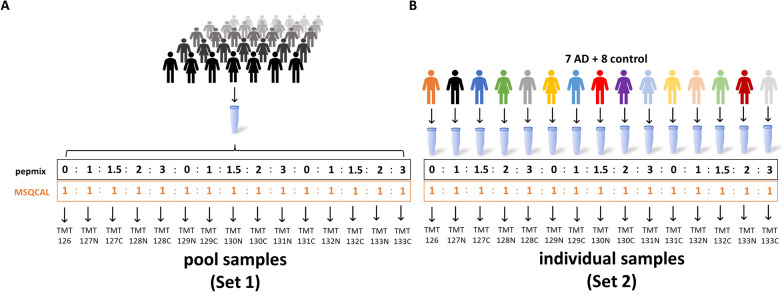
Experimental design to evaluate technical and total variation as well as an optimal normalization approach for our in-solution based TMT protocol. A pool of CSF from numerous individuals, referred to as Set 1 (**A**) or individual CSF samples consisting of 7 AD and 8 control samples, referred to as Set 2 (**B**) were split in 15 aliquots. Internal calibrator MSQCAL was spiked into each aliquot at equimolar amounts (50 fmol), while pepmix was added in differing amounts in triplicates (0, 10, 15, 20, 30 fmol). Each aliquot was subsequently TMT-labeled, combined, and analyzed by LC–MS

To evaluate both sources of variation in the in-solution TMT protocol and an optimal normalization strategy, an experimental design comprised of two TMT sets was chosen (Fig. 1). Briefly, one pool CSF sample was split in 15 aliquots. Each aliquot was spiked with an equimolar amount of a peptide mixture used for normalization (QCAL) and varying amounts (molar ratio 0:1:1.5:2:3) of a reference peptide mixture (pepmix) in triplicates. The samples were subsequently labeled and combined into the TMTpro 15-plex Set 1 (Fig. 1A). In the Set 2, CSF samples from 15 individuals (7 AD subjects and 8 controls) were prepared in the same manner (Fig. 1B). The calibrator QCAL served as reference standard for normalization to specific protein amount, hereafter referred to as QCAL normalization. Pepmix was used to analyze the performance of the normalization strategies and quantitation accuracy. Set 1 allowed us to determine the technical variation of the experiment, including sample preparation and MS analysis, while Set 2 mirrored the total variation of a real-life TMT experiment, made up of technical variation plus biological variation. Both AD and control samples were employed to portray more accurately a heterogeneous study group.

**Fig. 2:**
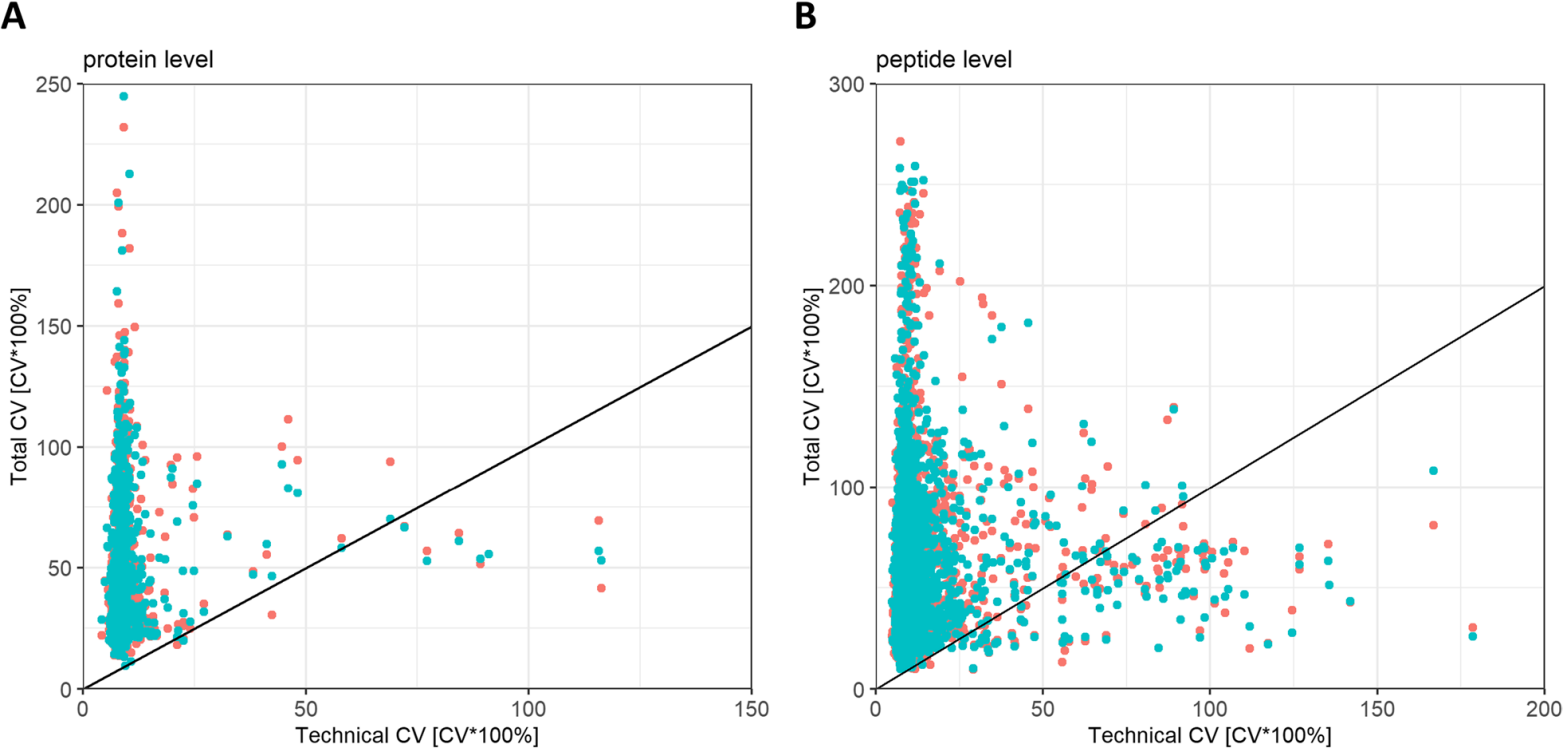
Coefficient of variation (CV) of proteins (**A**) and peptides (**B**) common to both Set 1 and Set 2. The technical CV was calculated based on data obtained from the pool CSF set (Set 1), while the total CV was obtained from the individual CSF sample data (Set 2). Each dot represents a protein (**A**) or peptide (**B**) measurement, respectively. The diagonally drawn line marks the threshold at which the total CV equals the technical CV. Duplicates of the Set 2 LC–MS analysis are color-coded (turquoise and red)

In the first step, intra-experimental variation was analyzed by determining the coefficient of variation (CV) of all proteins common to both pool samples and individual samples. The technical CV of each protein, calculated based on Set 1, was plotted against its corresponding total CV, as obtained from the individual samples (Set 2). No normalization was performed. CVs were evaluated at protein level, as well as at the peptide level (Fig. 2). Set 2 was divided into 2 aliquots, which were fractionated and independently analyzed via LC–MS. CVs from both runs are color-coded. Observations located left of the drawn line exhibit a lower technical CV than total CV, which applies to a great fraction of proteins and peptides, respectively. CVs were found to be largely independent of corresponding peptide and protein intensities (Additional file 2: Fig. S1). The data thus show that (i) the technical variation is comparatively small, with a median CV of appr. 10%, and that (ii) biological variation contributes substantially to the total variation we observe in TMT CSF experiments, even exceeding 200% for some proteins and peptides. Also, off-line fractionation as well as LC–MS analysis do not seem to introduce considerable variation as the CVs of both independent runs show great overlap. Overall, the considerably low technical variation attests to the validity of the method, facilitating the detection of biological differences.

### Comparison of normalization strategies using spiked-in protein standards

Next, we applied four normalization strategies to Set 1 and Set 2: (1) normalization to specific protein amount (QCAL normalization), (2) median normalization, (3) total peptide amount normalization and (4) quantile normalization. These normalization approaches were selected because they are relatively easy to implement and have shown promising results in previous studies [31, 32]. Especially median and quantile normalization have proven to yield good results in a comparative study evaluating normalization approaches in TMT proteomics [32]. Notably, normalization was performed on the protein level and was targeted at removing intra-experimental bias. Removal of inter-experimental bias (“batch effects”) will be further discussed towards the end of the paper.

**Fig. 3:**
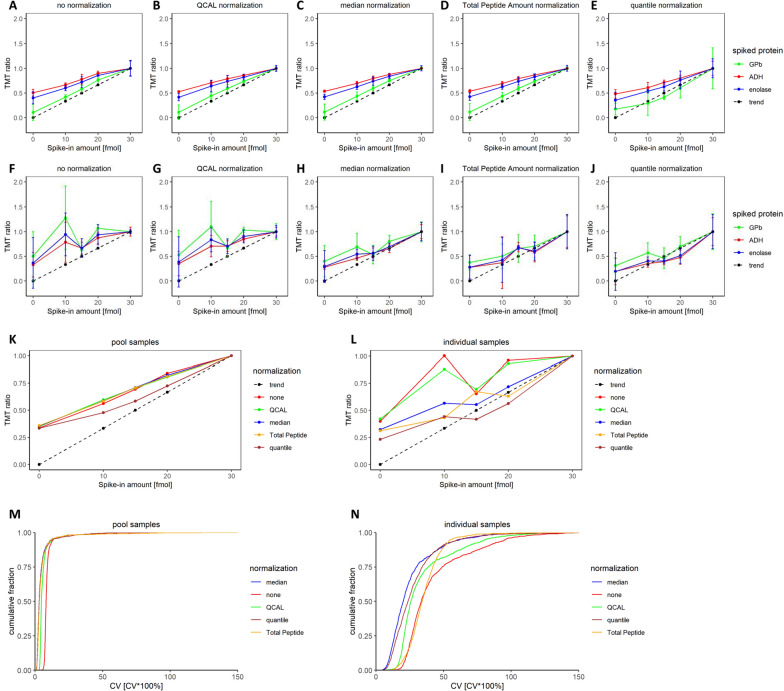
Performance of selected normalization approaches applied to a pool CSF (Set 1) and individual CSF TMT set (Set 2), as evaluated by pepmix TMT ratios and cumulative CV distribution. Following normalization of the pool CSF set (**A**–**E**) and individual CSF set (**F**–**J**), pepmix TMT ratios at each specified spike-in amount were evaluated separately for all three proteins GPb (green), ADH (red) and enolase (blue). Error bars indicate standard deviation for triplicate measurements. The dotted trendline represents the theoretically expected linear trend of TMT ratios. **K**, **L** Summarized mean TMT ratios of all 3 proteins plotted vs. their corresponding spike-in amount for selected normalization approaches (red: no norm., green: QCAL norm., blue: median norm., yellow: Total Peptide Amount norm., brown: quantile norm) applied to the pool CSF sample set (**K**) and individual CSF sample set (**L**). **M**, **N** Cumulative fraction of proteins below a certain CV in the pool samples set (**M**) and individual samples set (**N**) before and after normalization

Performance of the normalization approaches was evaluated by inspecting the TMT abundance ratios for three of the four proteins in pepmix (bovine serum albumin was excluded due to its extensive sequence overlap with human serum albumin): enolase, alcohol dehydrogenase (ADH) and glycogen phosphorylase b (GPb), versus their spike-in amount (Fig. 3A–J). In CSF pool samples (Set 1), only small differences were observed between any of the four normalization methods compared to the unnormalized data (Fig. 3A–E). For all proteins, the slope of the curve was slightly less than the expected trend (indicated by the dashed line in the plots), most likely owing to the phenomenon of ratio compression caused by co-isolation [18]. The similarity in performance between the methods is clearly visible when comparing the mean TMT ratios of all three proteins for each normalization approach as a function of spike-in amount (Fig. 3K), with quantile normalization differing slightly from the other methods and being closest to the expected trend.

While, against a homogeneous sample background, the effect of normalization was small, the abundance ratios in the dataset of the individual samples (Set 2) did not match the expected linear trend as closely (Fig. 3F–J). Quantitation accuracy seems to suffer from the heterogeneous sample background and the abundance ratios deviate from linearity, especially in the unnormalized dataset (Fig. 3F). The linearity of the correlation was, however, improved to varying extents by normalizing the data. While QCAL normalization had little to no effect on the ratios, median and quantile normalization mitigate deviation from linearity greatly (Fig. 3L). Normalization to total peptide amount as well as median and quantile normalization appeared to also improve the distortion towards the expected trend.

In addition, the cumulative fraction of all quantified proteins below a certain CV was analyzed for each normalization approach applied to the pool sample dataset (Fig. 3M) and individual samples dataset (Fig. 3N). Normalization of the pool samples dataset slightly decreased CVs, left-shifting the cumulative distribution curves. Again, all normalization approaches performed similarly. Hypothesizing that, despite high biological variation between CSF samples, most proteins should not vary greatly among individuals, cumulative distribution curves should also be left-shifted following successful normalization in Set 2. This is especially the case for median and quantile normalization which result in a noticeable decrease of protein CV.

Integrating the results, we thus conclude that (i) a heterogeneous sample background, such as the varying sample composition of CSF samples from different individuals, negatively affects quantitation accuracy, and that (ii) normalization can improve abundance ratios considerably in biologically diverse TMT sets. The plots show that median and quantile normalization appear to be most successful in reducing overall CVs in Set 2, albeit quantile normalization may overcorrect the data as indicated by TMT ratios dipping below the expected linear trend.

**Fig. 4:**
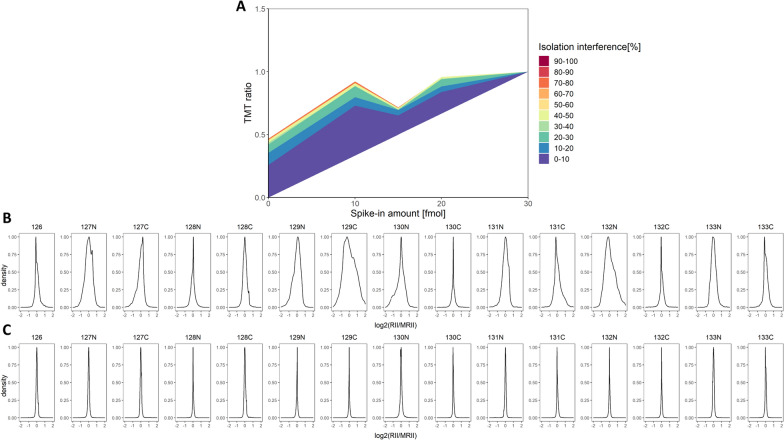
Influence of co-isolation on pepmix TMT ratios and peptide abundance distribution in both individual and pool CSF TMT sets. Mean pepmix TMT ratios were evaluated based on the extent of co-isolation in the corresponding peptide spectrum match. The data was plotted in a cumulative fashion displaying the mean TMT ratio of the fraction of pepmix data below the respective co-isolation threshold (**A**). Median-centered peptide abundance distribution for each TMT channel (sample) in (**B**) the TMT set comprised of individual samples and (**C**) the TMT set consisting of pool CSF samples

Encouraged by the clear difference in quantitation accuracy between pool and individual samples, we set out to determine the underlying reason. We hypothesized that the observed deviation from linearity in the presence of a heterogeneous sample background may be attributable to co-isolation during precursor selection. In the TMT set comprised of individual CSF samples, TMT channel reporter ion intensities can be affected to varying extents by co-isolation. The effect is dependent on the presence and abundance of the co-isolating peptide in each respective TMT channel and can thus distort TMT ratios to differing degrees. Meanwhile, in a TMT set with a homogeneous sample background, co-isolated peptides are expected to contribute equally to each TMT channel, so that the linear trend can be retained. Due to varying peptide abundance distributions of individual CSF samples (Fig. 4B) as opposed to pooled CSF samples (Fig. 4C), differential effects may be quite pronounced.

Indeed, this is what could be observed when evaluating pepmix abundance ratios at different co-isolation thresholds. It is apparent that co-isolation negatively affects linearity in a heterogenous sample background (Fig. 4A). TMT ratios are not only progressively distorted towards a 1:1 ratio but also deviate from linearity increasingly.

### Comparison of MS2 and MS3 quantification accuracy

**Fig. 5:**
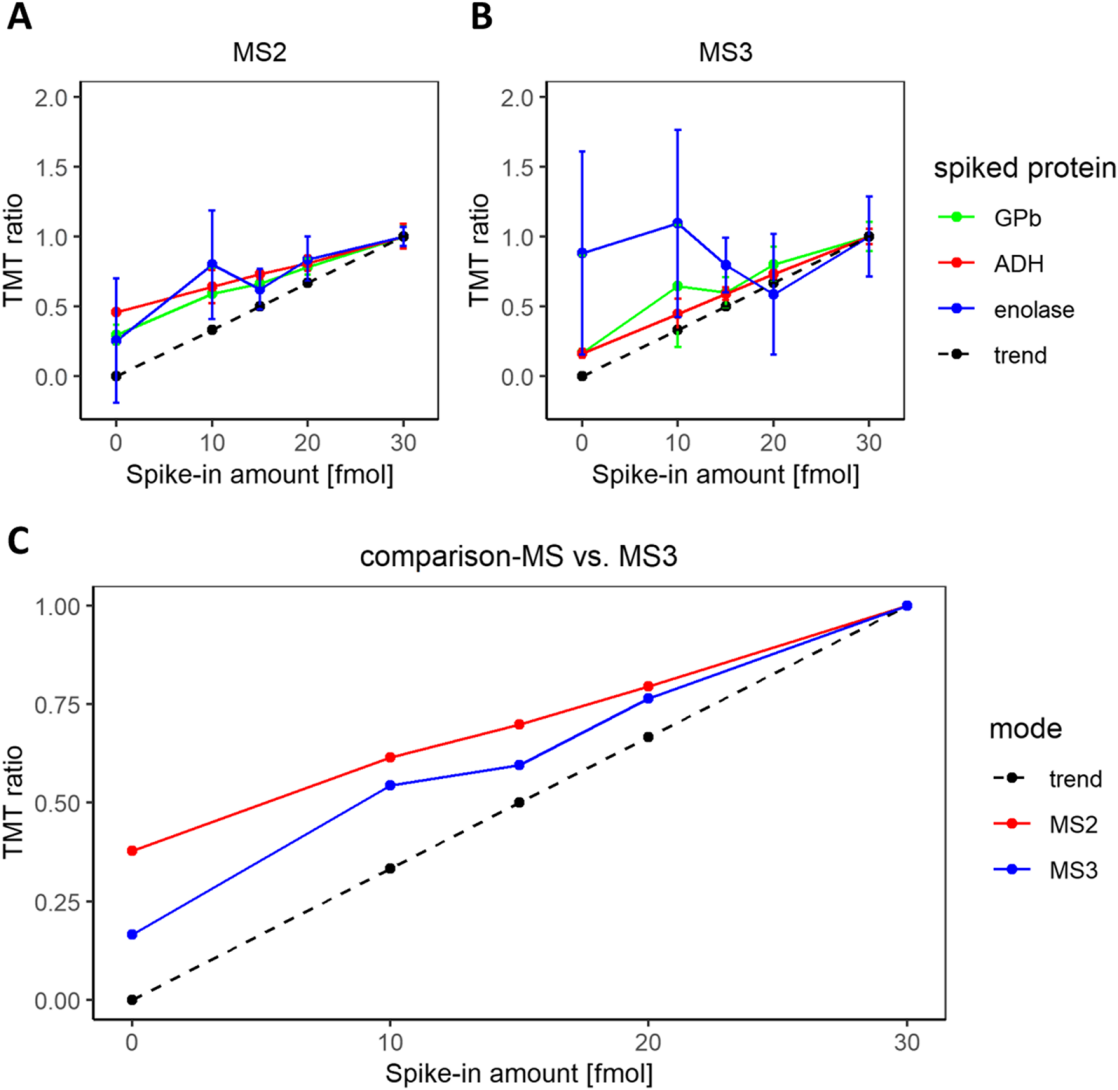
Measured TMT ratios of spiked pepmix proteins in MS2 and MS3 mode. Pepmix TMT ratios at each specified spike-in amount were evaluated separately for all three proteins P00489 (GPb, green), P00330 (ADH, red) and P00924 (enolase, blue) in MS2 (**A**) and MS3 (**B**) mode. Error bars indicate standard deviation for triplicate measurements. The dotted trendline represents the theoretically expected linear trend of TMT ratios. **C** Summarized mean TMT ratios of P00489 and P00330 plotted against their corresponding spike-in amount (unnormalized data). TMT ratios measured in MS2 mode are colored in red and TMT ratios measured in MS3 mode are depicted in blue

For comparison of MS2 and MS3 quantitation accuracy, Set 2 was analyzed in both MS2 mode and with SPS MultiNotch MS3 [18], consecutively. To assess the extent of ratio compression, TMT ratios of the spiked pepmix proteins were evaluated at each corresponding spike-in amount (Fig. 5). In the MS2 mode, the curves for all three proteins followed a linear trend quite closely (Fig. 5A). Fitting a linear regression to the data, the slope was lower than that of the expected trendline, which points to ratio compression.

In the MS3 mode, both ADH and GPb abundance ratios are closer to the expected ratio values than in MS2 (Fig. 5B). As expected, MS3 can mitigate the effects of ratio compression observed in MS2. However, enolase exhibits heavily distorted TMT ratios, with only one peptide quantified. This occurred most likely because of a decreased detection sensitivity in MS3 and because enolase is less abundant in the pepmix compared to the other proteins. Mean abundance ratios of the proteins ADH and GPb, confidently quantified in both MS2 and MS3 mode, were plotted against the corresponding spike-in amount (Fig. 5C). Directly comparing both analysis modes, ratio compression is indeed reduced in MS3. However, 1242 proteins could be identified in MS2 mode while only 886 proteins (71%) were detected with MS3. Both observations are in line with previous studies [33, 34].

### Evaluating the performance of sample preparation techniques

To assess the suitability of FASP as sample preparation technique for CSF, we compared it to the in-solution protocol. In FASP, the sample is loaded on a 30 kDa molecular cut-off filter, on which all reaction steps are performed, and reagents can be removed by centrifugation, while the proteins are retained on the filter. This permits usage of strong detergents potentially increasing the number of identified proteins and eliminates matrix effects.

**Fig. 6:**
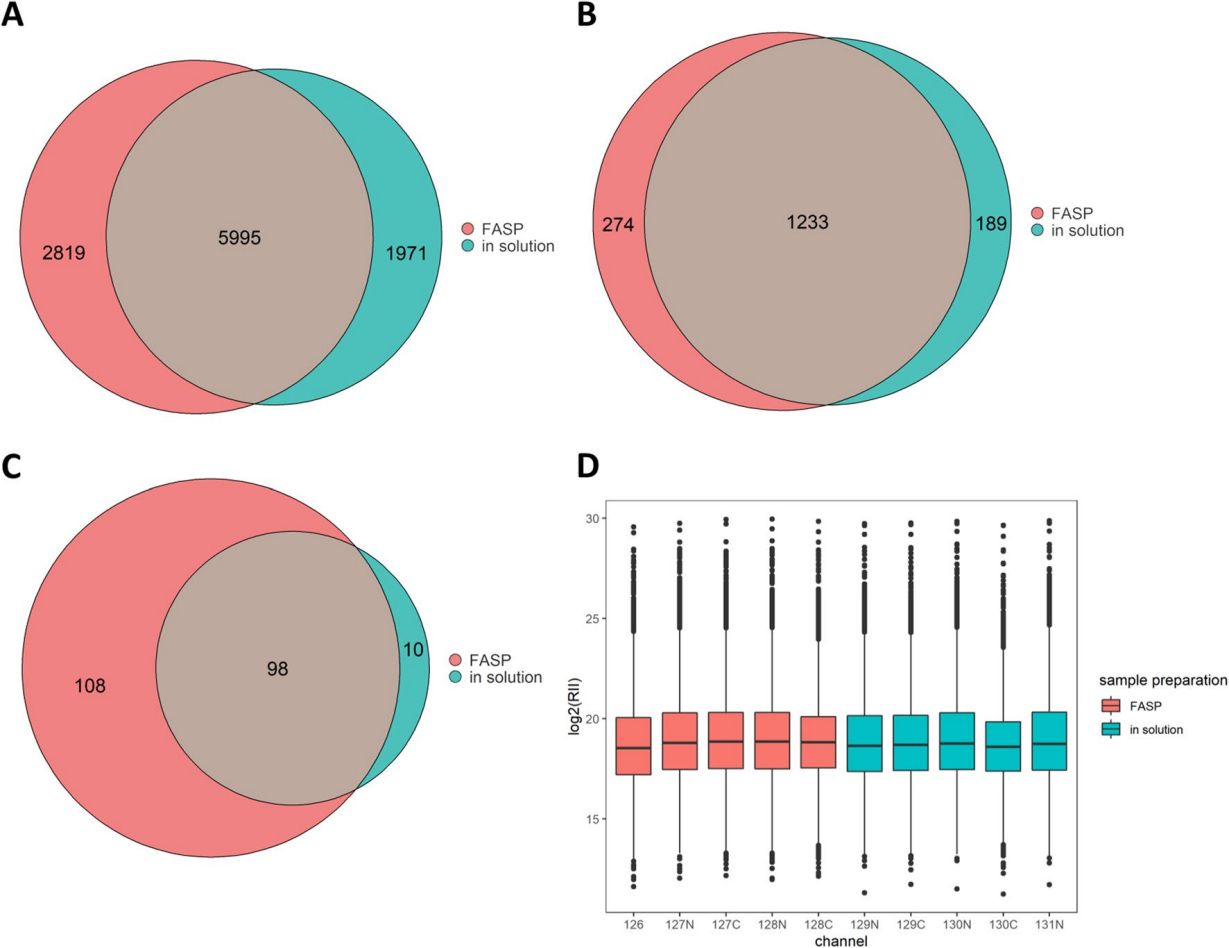
Comparison of the sample preparation techniques (1) in-solution digestion and (2) filter-assisted sample preparation (FASP). Five replicate CSF pool samples were prepared with in-solution digestion protocol and FASP each. LC–MS analysis was performed separately for both sets of replicates. Following quantification, the number of peptides identified employing FASP and in-solution digestion were determined (**A**). Likewise, the amount of identified proteins with both approaches was evaluated (**B**). To assess contamination, the number of peptides derived from keratins was calculated for the two preparation techniques (**C**). Log2-transformed reporter ion intensities (RII) of peptide group abundances were plotted for all TMT channels (in-solution and FASP) for evaluation of yield and reproducibility (**D**)

We prepared CSF pool samples employing both in-solution and FASP protocols in technical replicates of five, respectively. The replicates were labeled and separately combined into two TMT sets (one FASP set, one in-solution set), which were fractionated and analyzed independently. A total of 5995 peptide groups were commonly identified in both sets, while an additional 1971 peptide groups were exclusively detected in the in-solution set and 2819 in the FASP set (Fig. 6A). On the protein level, 1233 proteins were detected with both preparation techniques. Employing the FASP technique, an additional 274 proteins could be identified as compared to an additional 189 proteins with the in-solution protocol (Fig. 6B).

While the FASP protocol allowed identification of more proteins and peptide groups than in-solution digestion, 216 of the peptide groups identified with FASP were keratins, a commonly observed contaminant in proteomic experiments, originating from skin, hair and nails [35]. In comparison, only 108 keratin peptide groups were identified with the in-solution protocol (Fig. 6C). These results indicate that the FASP protocol may be more prone to contamination from the laboratory environment than the in-solution protocol.

Since we were also concerned with reproducibility and peptide yield, we assessed the peptide group abundance of each channel in both sets. Log-transformed peptide group abundances neither differed largely between the TMT channels nor between sample preparation techniques (Fig. 5D), indicating similar peptide yield for the protocols. To determine reproducibility of technical replicates, the median CV of the protein abundances was calculated, resulting in 4.3% for in-solution and 10.8% for FASP (unnormalized data). Consequently, in-solution appears to be more reproducible than FASP. Lastly, TMT labeling efficiencies of the two approaches were compared. With a labeling efficiency of 98% for FASP and 95% for in-solution digestion, the sample preparation techniques both allow for near-complete labeling.

### TMT labeling efficiency

To determine the minimum TMT amount required for efficient labeling of CSF proteins, individual CSF samples were labeled with different amounts of the TMTpro reagents: 0.5 mg, 0.25 mg and 0.1 mg of TMT were employed per labeling reaction of 50 µL CSF (containing an estimated 15–20 µg of protein) resulting in three TMT sets. The TMT sets were then fractionated and analyzed separately. Labeling efficiencies as well as median peptide and protein CVs were calculated for each set (Table 1). The labeling efficiency was determined by subtracting the amount of peptide groups without a TMT label from the ones with TMT label, divided by the sum of all peptide groups. Decreasing the TMT amount by half reduces labeling efficiency by a mere 3.5% while a fivefold reduction in TMT amount leads to a 10% decrease in labeling efficiency. Neither median peptide nor protein CV seem to be affected by a decrease in TMT amount down to 0.1 mg/50 µL CSF.

**Table 1:** Parameters evaluating the completeness of the TMT labeling reaction at 0.5 mg, 0.25 mg, and 0.1 mg per 50 µL CSF sample

Amount TMT/50 µL CSF [mg]	Labeling efficiency [%]	Median peptide CV [%]	Median protein CV [%]	Median log2(MRII)
0.5	91.42	43.9	36.9	16.2
0.25	87.93	47.5	36.1	14.5
0.1	79.98	45.5	34.6	15.7

As incomplete labeling is expected to coincide with an overall decreased reporter ion intensity (RII), RII was evaluated for each channel in each set (Additional file 3: Fig. S2). Comparing all 3 sets, a slight decrease in the median RII (MRII) can be observed if less than 0.5 mg TMT is employed (Table 1). However, log2 (MRII) is higher for 0.1 mg than for 0.25 mg TMT. In conclusion, no clear trend could be observed pointing to incomplete labeling.

Next, the distribution of median centred peptide ratios of peptides common to all sets was investigated to determine whether reduced TMT amount could distort peptide ratio distributions. Distributions were plotted for each sample (channel) across all sets (Additional file 4: Fig. S3). In most samples, peptide ratio distributions are not affected by a decreased TMT amount. Some of the 0.25-mg TMT samples (127N, 127C, 129C) and/or 0.1 mg TMT samples (129C, 130C, 132N, 133C) displayed a slightly altered distribution but overall, the RII distributions were similar for samples labeled with different amounts of TMT.

In conclusion, labeling efficiency continuously decreases with decreasing TMT amount. However, the data indicate that MRII and peptide ratio distribution are only slightly, if at all, affected by the reduction in TMT amount.

### Performance of the normalization methods in a clinical study of AD

We applied all four normalization methods to a published data set from a clinical proteomic study of a cohort from the European Medical Information Framework (EMIF), comprising CSF from AD patients (n = 93), healthy controls (n = 126), and patients with mild cognitive impairment (n = 198) as well as subjective cognitive impairment (n = 61): median normalization, normalization to total peptide amount, quantile normalization and the TAMPOR function, which has been recently employed in numerous TMT CSF studies [24, 25, 36]. The TAMPOR function can be viewed as an extension of median normalization and is essentially based on Tukey’s median polish algorithm. It aims at removing batch effects while remaining robust to outliers and preserving overall biological variance [24]. Removal of batch effects *i.e.*, inter-experimental bias, is important when extracting data from multiple TMT sets. It is often achieved by the addition of a GIS to each set, comprised of pooled individual study samples [37]. The GIS channel serves as reference sample and can be used as denominator when calculating protein-wise ratios.

**Fig. 7:**
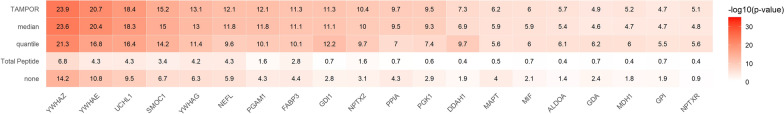
Heatmap of BH-corrected p-values for the top 20 biomarker candidates identified with all normalization approaches

To assess the performance of the normalization methods, we examined potential biomarker candidates with protein abundances significantly altered in AD subjects vs. controls and tested how the significance of the association of these proteins with clinical AD was influenced by the respective normalization method. In addition, we investigated how many biomarker candidates could be identified with each normalization approach and how this compared to the unnormalized data. Importantly, abundance differences between control and AD subjects are expected to be strengthened for true biomarkers upon successful normalization, resulting in improved p-values and thus more biomarker candidates. Using t-test and applying BH-correction for multiple testing, a total of 64 biomarker candidates were identified using TAMPOR, closely followed by quantile normalization (63) and median normalization (61). In contrast, total peptide amount normalization and unnormalized data yielded 7 and 19 potential candidates, respectively. Comparing the corrected p-values of the top 20 biomarker candidates among normalization approaches, mean p-values were lowest for quantile (5.3 × 10^–9^), TAMPOR (4.5 × 10^–8^) and median (5.0 × 10^–8^) (Fig. 7). TAMPOR and median performed especially well for the top 12 candidates while for the top 13–20 candidates, quantile normalization yielded the lowest p-values. Total peptide normalization performed worse than no normalization, yielding considerably higher p-values than all other approaches evaluated.

Using TAMPOR normalization, we extracted a list of 64 AD biomarker candidates, 46 of which were increased and 18 of which were decreased in AD subjects compared to cognitively healthy controls (Table 2). Cross validating these protein hits with two recent independent studies by Higginbotham et al. and Bader et al., we discovered significant overlap among proposed biomarker candidates [36, 38]. Out of the 64 biomarker candidates, 23 proteins (36%) were found to also be significantly increased in the CSF1 discovery cohort of Higginbotham (FDR < 1%), while 14 proteins (22%) overlapped with the “40 protein signature of AD” put forward by Bader [38].

**Table 2:** List of top 64 proteins significantly differing in abundance between AD and control subjects in the EMIF-AD cohort

Accession number	Gene name	BH-corrected p-value	log2-fold change	Number of observations control	Number of observations AD
P63104	YWHAZ	1.4E−24	0.49	122	91
P62258	YWHAE	1.8E−21	0.37	122	91
P09936	UCHL1	4.1E−19	0.37	106	77
Q9H4F8	SMOC1	5.8E−16	0.31	120	90
P61981	YWHAG	7.2E−14	0.31	109	83
P07196	NEFL	7.5E−13	0.58	69	52
P18669	PGAM1	7.5E−13	0.20	109	84
P05413	FABP3	5.6E−12	0.29	112	82
P31150	GDI1	5.6E−12	0.17	123	88
P47972	NPTX2	4.4E−11	−0.35	125	91
P62937	PPIA	1.8E−10	0.20	121	89
P00558	PGK1	3.0E−10	0.15	114	90
O94760	DDAH1	4.9E−08	0.18	124	93
P10636	MAPT	6.9E−07	0.40	56	45
P14174	MIF	9.7E−07	0.19	101	82
P04075	ALDOA	2.2E−06	0.19	124	90
P62942	FKBP1A	2.5E−06	0.21	105	78
Q92686	NRGN	4.3E−06	0.31	99	73
P16949	STMN1	5.8E−06	0.20	118	87
P40925	MDH1	6.0E−06	0.19	125	89
P33908	MAN1A1	6.0E−06	− 0.08	122	91
O95502	NPTXR	7.5E−06	− 0.24	126	91
Q6EMK4	VASN	1.2E−05	0.10	123	91
Q9Y2T3	GDA	1.4E−05	0.24	125	93
P12277	CKB	1.8E−05	0.14	120	90
Q12805	EFEMP1	1.8E−05	0.12	125	91
P36222	CHI3L1	2.2E−05	0.26	124	92
P06744	GPI	2.2E−05	0.21	73	67
P00492	HPRT1	2.4E−05	0.20	119	87
Q92765	FRZB	2.4E−05	0.20	125	92
O15240	VGF	2.7E−05	− 0.26	124	90
P60983	GMFB	3.9E−05	0.11	121	89
Q13421	MSLN	7.3E−05	− 0.23	121	89
O00142	TK2	2.6E−04	0.13	107	81
Q13228	SELENBP1	2.7E−04	− 0.15	124	90
P23297	S100A1	3.0E−04	0.22	48	33
P17677	GAP43	3.0E−04	0.19	126	90
Q92743	HTRA1	3.1E−04	0.09	126	92
P0DP23	CALM1	3.7E−04	0.16	120	89
P06733	ENO1	3.8E−04	0.14	119	89
P31946	YWHAB	3.9E−04	0.17	116	90
Q9HCB6	SPON1	4.2E−04	0.11	125	91
P62328	TMSB4X	4.6E−04	0.15	124	90
P10451	SPP1	6.1E−04	0.16	126	90
Q9Y279	VSIG4	6.3E−04	0.17	123	88
P29120	PCSK1	6.3E−04	− 0.22	121	90
P14618	PKM	7.4E−04	0.15	119	90
P10599	TXN	8.0E−04	0.11	121	89
P05060	CHGB	8.6E−04	− 0.14	125	90
P55286	CDH8	1.5E−03	− 0.11	124	91
Q14697	GANAB	1.6E−03	− 0.09	124	89
P01213	PDYN	1.8E−03	− 0.14	123	91
Q06830	PRDX1	1.9E−03	0.10	120	87
Q5VSG8	MANEAL	2.1E−03	− 0.10	124	90
Q9H2A7	CXCL16	2.7E−03	0.16	85	63
P08637	FCGR3A	2.8E−03	0.16	111	88
P54756	EPHA5	3.2E−03	− 0.13	126	93
O60241	ADGRB2	3.5E−03	− 0.13	121	90
P09104	ENO2	3.6E−03	0.12	125	89
Q92932	PTPRN2	3.8E−03	− 0.13	125	93
P13521	SCG2	4.5E−03	− 0.16	123	91
P01303	NPY	4.6E−03	− 0.24	99	71
P30086	PEBP1	5.3E−03	0.10	123	91
P01210	PENK	6.3E−03	− 0.12	121	90

Data was normalized employing the TAMPOR function

**Fig. 8:**
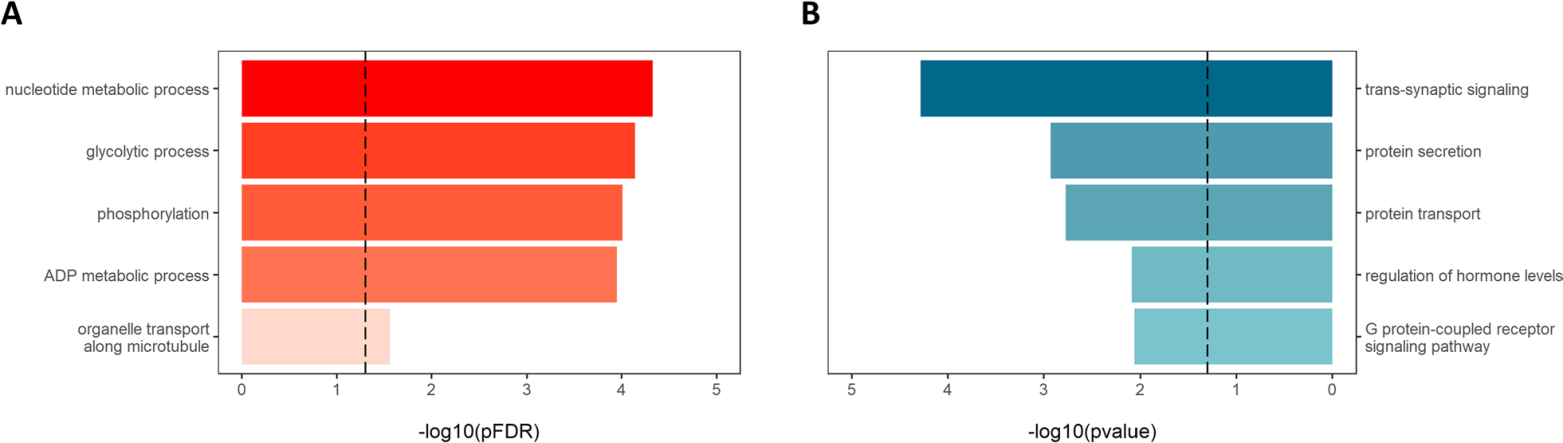
Statistical overrepresentation test of GO-terms enriched in significantly increased (**A**) and significantly decreased proteins (**B**) in AD subjects vs. controls. Of all significantly enriched GO-terms, 5 terms were chosen that ranked among the most significantly enriched and best represented the entire list. The vertically drawn dashed line marks the threshold of p < 0.05. In the case of significantly increased proteins, an FDR correction was performed while for the decreased proteins uncorrected p-values are displayed

Finally, we conducted a gene ontology (GO) analysis to determine biological processes enriched in the group of significantly increased and decreased proteins, respectively. GO terms ranking among the most significant, and best portraying the obtained list of GO hits were selected (Fig. 8). The entire list of GO terms is available as Additional file 1. Proteins significantly increased in AD mostly mapped to glycolytic-related processes as well as core metabolic processes such as energy and nucleotide metabolism. In addition, multiple proteins were linked to phosphorylation, a post-translational modification implicated in abnormal tau accumulation [39].

In contrast, the list of significantly decreased proteins was too small to apply a stringent BH-based cut-off. Instead, uncorrected p-values were evaluated. Decreased proteins were mainly enriched in GO-terms relating to synaptic signalling or signalling pathways in general. Further, protein secretion and hormonal regulation were found to be among the top enriched GO-terms.

## Discussion

### Normalization of TMT ratios in CSF

A general recommendation for TMT proteomic analysis is that the overall protein composition and concentrations of the samples is kept similar, to ensure even labeling yield and similar matrix effects between samples. Therefore, in TMT analysis of cell and tissue samples, the protein concentration in the study samples is determined and adjusted so that the same amount is subjected to TMT labeling. This strategy, however, would arguably be unlikely to be successful for CSF, in which protein composition and concentration of CSF varies significantly between individuals. A major contributor is blood–brain barrier function, which decreases in several medical conditions including AD, leading to a greater influx of blood proteins into the CSF [40, 41]. Brain atrophy, occurring in many neurodegenerative disorders, leads to decreased general secretion of proteins from the affected regions. Moreover, differing CSF turnover rates may affect CSF protein concentrations [42, 43]. Measurement of specific biomarkers relative to the total protein concentration in CSF would thus be strongly distorted by such general variations. This is a well-known fact in clinical laboratory medicine, where established CSF markers of neurodegenerative pathologies, such as the amyloid-β 1–42/1–40 ratio, total-Tau, phospho-Tau, neurofilament-light polypeptide, and glial-fibrillary acidic protein, are all measured relative to CSF volume and not protein concentration [44–46], as the latter would degrade their biomarker performance significantly.

A potential drawback of performing TMT proteomic analysis with fix-volume CSF samples is that differences in protein concentration and composition of the samples may distort quantification. Furthermore, compositional bias may also affect the performance of the normalization methods used to adjust the TMT reporter ion intensities to correct for systematic experimental variation. Indeed, this is what we observed when comparing TMT quantification of a set of test proteins prepared in aliquots of a CSF pool or in individual CSF samples (Fig. 3). For the CSF pool (identical protein composition and concentrations in all samples), the TMT ratios of all test proteins showed a strong linear response relative to their concentration, and all four tested normalization methods performed similarly. In contrast, for the individual samples, the correlation between TMT ratios and concentrations of the test proteins was worse, and the choice of normalization method affected the result more. We hypothesize that this discrepancy in quantitation accuracy may be due to interferences from co-isolated peptides which, depending on individual sample composition and peptide abundance, affect the reporter ion intensities to varying degrees.

Normalization to a spiked-in peptide standard (QCAL) showed the lowest performance of the tested normalization strategies, yielding results similar to those obtained without normalization. This is in agreement with a TMT plasma study conducted by Dubois et al. [32] in which normalization to an external standard protein yielded comparable results to unnormalized data. As normalization to an external spike-in is targeted at minimizing technical variation, it may be argued that the present data already exhibits low technical variability and that consequently results cannot be improved significantly by normalization to a spiked-in peptide standard. This is indeed supported by data displayed in Fig. 2, showing limited technical variation. QCAL, being a non-human peptide mix added before sample preparation, can neither correct for unwanted biological variability nor potential variation in tryptic digestion or pre-analytical variability. Since unwanted biological variability may arguably be the greatest factor influencing quantitation, an external spike-in standard appears rather unsuitable for TMT CSF.

Median and quantile normalization appear to have outperformed Total Peptide amount normalization in that they were more successful in reducing overall CVs (Fig. 3N). One may speculate that this is because median- and quantile methods are based on normalizing TMT ratios relative to median and overall sample distribution rather than total protein abundance, making them less liable to aberrant abundances in a few proteins, even if these are large. In addition, both median and quantile normalization make individual TMT channels more comparable by either shifting or equalizing abundance distributions across a TMT set. Likely, this leads to a partial correction for unwanted biological variability resulting in a reduction of overall CV.

### Comparison of MS2 and MS3 quantification

Comparing TMT ratios obtained with MS2 and MS3 mode shows that MS3 can mitigate the effects of ratio compression. However, as expected, this comes at the expense of detection sensitivity leading to a smaller number of proteins which can be identified in an exploratory study. Introducing a third stage of precursor isolation and fragmentation leads to the loss of reporter ion intensities at MS3 level which coincides with a reduced number of quantifiable peptides. We thus recommend that both competing aspects, detection sensitivity and accuracy, are carefully weighed in the face of the purpose of the respective study. For exploratory purposes, for instance, it may be advisable to employ MS2 to maximize the number of identified proteins, forfeiting quantitation accuracy.

### Evaluating the performance of sample preparation techniques

Comparative evaluation of an in-solution sample preparation protocol with FASP demonstrated that (i) more peptides and proteins could be detected with the FASP approach but also that (ii) FASP appears to be more liable to contamination from the laboratory environment, as indicated by the identification of larger number of keratin-derived peptides. Peptide yield and labeling efficiency did not differ considerably among both approaches. Higher sensitivity of FASP towards contamination may be explained by the multitude of sample preparation and spinning steps, each of which increases the likelihood for contamination and allows for contaminants on the lid tube to be transferred to the bottom. Most likely, more proteins were identified with FASP due to the usage of SDS as strong ionic detergent which efficiently solubilizes and denatures proteins in CSF. Despite the high number of identified proteins employing FASP, it should be noted that it is more laborious than in-solution and requires molecular weight cut-off filters. This can pose a major drawback, especially when conducting large-scale proteomic studies.

### TMT labeling efficiency

TMT labeling efficiency analysis revealed that labeling efficiency is decreased by appr. 10% when reducing the TMT amount by 80%. Although this does appear like a considerable reduction in labeling efficiency, neither MRII nor sample peptide distribution seemed to be affected largely. Thus, it can be inferred that a decrease in TMT amount of up to 0.1 mg/50 µL CSF does not influence the experimental outcome to a large extent. The official TMT labeling protocol recommends a ratio range of 1:5 to 1:10 (w:w), sample to tag [47]. However, this value is not necessarily expected to be applicable to crude CSF with a severely complex sample background. Assuming the protein amount of 7.5 µg to 30 µg per 50 µL CSF [10, 11], this would correspond to a minimum TMT amount of 0.04–0.15 mg, respectively. All in all, it appears that the sample background does not significantly interfere with the TMT labeling reaction and that TMT protocol recommendations can be followed.

### Application to EMIF-AD dataset

Application of different normalizations to the EMIF-AD dataset showed that median-based approaches (TAMPOR and median) as well as quantile normalization outperform no normalization and normalization based on total peptide amount. As suggested above, median-based and quantile normalization may be superior to total peptide normalization as they are more robust to outliers i.e., highly abundant proteins, and are superior in maximizing the comparability among TMT channels. In fact, our data show that even no normalization is preferable to normalization to total peptide amount.

TAMPOR, following a median polish algorithm, appears to yield slightly better p-values than the simple median shift. This may indicate that dedicated algorithms are required to optimally handle CSF sample complexity and to adequately control for batch effects. Overall, TAMPOR, median and quantile normalization performed quite similarly. Comparison of our results with available literature further supported the validity of our normalization approaches. However, it should be noted that quantile normalization forces all samples to adopt a uniform abundance distribution which could potentially obscure biological differences and overcorrect the data. We thus conclude that likely median-based normalization appears most suitable for CSF TMT data.

Notably, data normalization was mainly conducted on the protein level in this study. Previous studies have shown that normalization can also be successfully applied at spectrum or peptide level [48–50]. The choice depends on several factors including the software used for MS data processing as well as the generated output. Also, the extent of analytical bias introduced by downstream data processing, such as rolling up peptide to protein abundances, may play a role. Importantly, only a fraction of all available and popular normalization techniques in TMT has been tested in this study [51], making no claim to completeness. Thus, it cannot be excluded that even more suitable normalization approaches may exist for TMT CSF data.

By employing the TAMPOR function, we were able to identify 64 potential biomarker candidates significantly altered in AD patients compared to cognitively healthy controls in the EMIF cohort. We investigated enriched GO terms among these proteins and found significantly increased proteins in AD to be mainly related to core metabolic as well as glycolytic processes. To date, mounting evidence indeed suggests a deregulation in both energy and purine metabolism in AD subjects [52–55]. Also, glycolytic dysfunction has long been recognized to occur even in the preclinical stage of AD [56]. Significantly decreased proteins, on the other hand, were largely enriched in GO-terms mapping to synaptic signalling, protein secretion or transport. According to many studies, synaptic dysfunction is a key characteristic of AD [57]: loss of synapses positively correlates with AD severity. It thus appears that enriched GO-terms are congruent with AD-related findings stated in the literature, providing additional indication for their involvement in AD pathology.

## Conclusion

In this study we have addressed multiple challenges associated with TMT proteomics in CSF. We ascertained that a heterogeneous CSF sample background negatively affects quantitation accuracy and necessitates appropriate normalization. While normalization to an external spiked-in peptide standard, as well as normalization to total peptide amount, yielded unsatisfactory results, median-based approaches and quantile normalization evidently reduced unwanted variability. In addition, we generated data to help evaluate the choice of adequate TMT amount, sample preparation technique as well as MS analysis mode for respective TMT studies in CSF. Finally, we presented a biomarker candidate list of 64 significantly altered proteins in the proteome of AD patients compared to controls stemming from the EMIF-cohort.

Looking to the future, we hope that our results can provide guidance when working with TMT in CSF samples and similar clinical studies where heterogeneous samples are involved, helping to make critical decisions for sample preparation and data analysis. In addition, we are confident that the biomarker candidate list presented in this study can form the basis for future biomarker development studies.

## Supplementary Information

**Additional file 1.** List of complete GO-terms relating to both significantly increased and decreased proteins (clinical AD vs. control) in EMIF-AD. Description of data: GO biological process terms were generated with the PANTHER statistical overrepresentation test. For significantly increased proteins, an FDR-correction was performed.

**Additional file 2: Figure S1.** Dependency of CV on protein and peptide intensities in both pool (set 1) and individual CSF sample sets (set 2). Technical CVs were plotted vs. their corresponding log-transformed protein (A) and peptide intensities (B) in the pool CSF dataset. Likewise, the dependency of total CVs on log-transformed protein (C) and peptide (D) intensities was evaluated for the individual CSF sample dataset.

**Additional file 3: Figure S2.** Reporter ion intensities (RII) per TMT channel in log2-space at differing TMT amounts. For each individual CSF sample corresponding to a specific TMT channel, peptide RIIs were calculated, log2-transformed, and visualized as boxplot. Every sample was labeled with 3 different TMT amounts per 50 µL CSF: 0.5 mg, 0.25 mg, and 0.1 mg.

**Additional file 4: Figure S3.** Distribution of median centred peptide-ratios for each individual CSF sample i.e., channel at differing TMT labeling amounts. Individual CSF samples, corresponding to a specific TMT channel, were prepared separately, and labeled with 0.5 mg (blue), 0.25 mg (black), 0.1 mg (red) TMT per 50 µl CSF. Following LC–MS analysis, the density distribution of log2 peptide ratios (channel reporter ion intensity) was calculated and median centred employing median reporter ion intensity in each sample (MRII). Only peptides common to all three sets were considered for analysis.

## Data Availability

The datasets used and/or analysed during the current study are available from the corresponding author on reasonable request.

## Abbreviations

ACN: Acetonitrile
AD: Alzheimer’s disease
ADH: Alcohol dehydrogenase
BH: Banjamini and Hochberg
BSA: Bovine serum albumin
CSF: Cerebrospinal fluid
CV: Coefficient of variation
DTT: Dithiothreitol
EMIF: European Medical Information Framework
EMIF-AD MBD: European Medical Information Framework Alzheimer’s Disease Multimodal Biomarker Discovery
FASP: Filter-assisted sample preparation
FDR: False discovery rate
GIS: Global internal standard
GO: Gene ontology
GPb: Glycogen phosphorylase b
HA: Hydroxylamine
IAA: Iodoacetamide
LC–MS: Liquid chromatography coupled to mass-spectrometry
MRII: Median reporter ion intensity
MS: Mass spectrometry
pepmix: Reference peptide mix
RII: Reporter ion intensity
RII: Reporter ion intensity
SDC: Sodium deoxycholate
SDS: Sodium dodecyl sulfate
SPE: Solid phase extraction
SPS: Synchronous precursor selection
TEAB: Triethylammonium bicarbonate
TFA: Trifluoroacetic acid
TMT: Tandem mass tag
