# Supplementary figures and images for: Optimized sample preparation and data analysis for TMT proteomic analysis of cerebrospinal fluid applied to the identification of Alzheimer’s disease biomarkers
Source: Clin Proteomics. 2022 May 14;19:13. doi: 10.1186/s12014-022-09354-0 (PMC9107710; doi:10.1186/s12014-022-09354-0)

**A**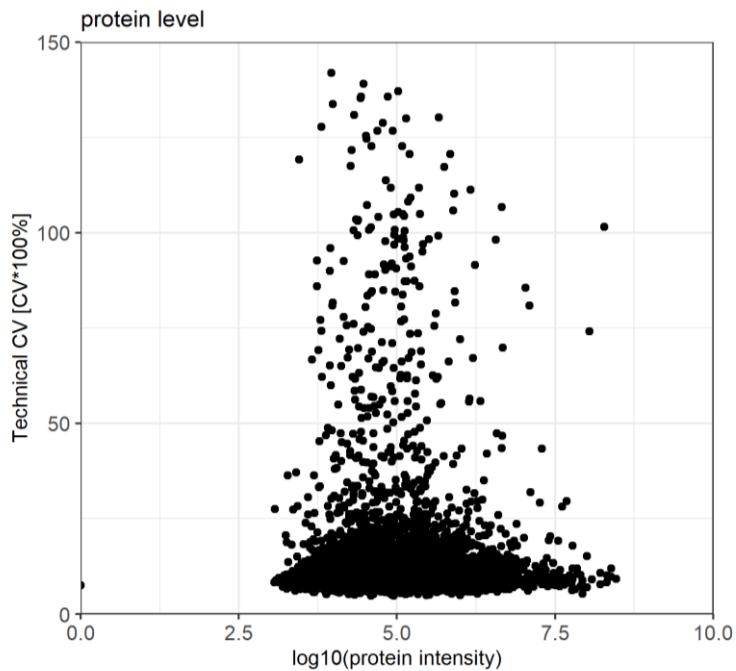**B**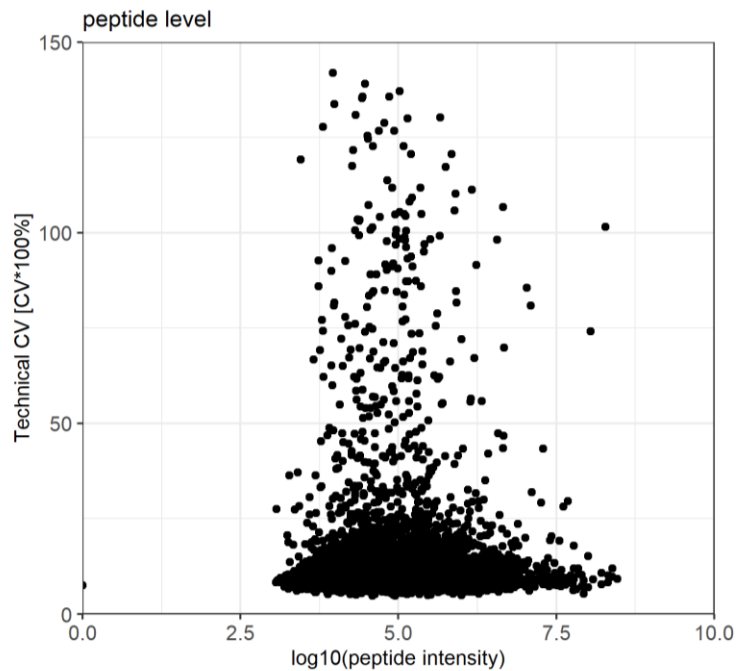**C**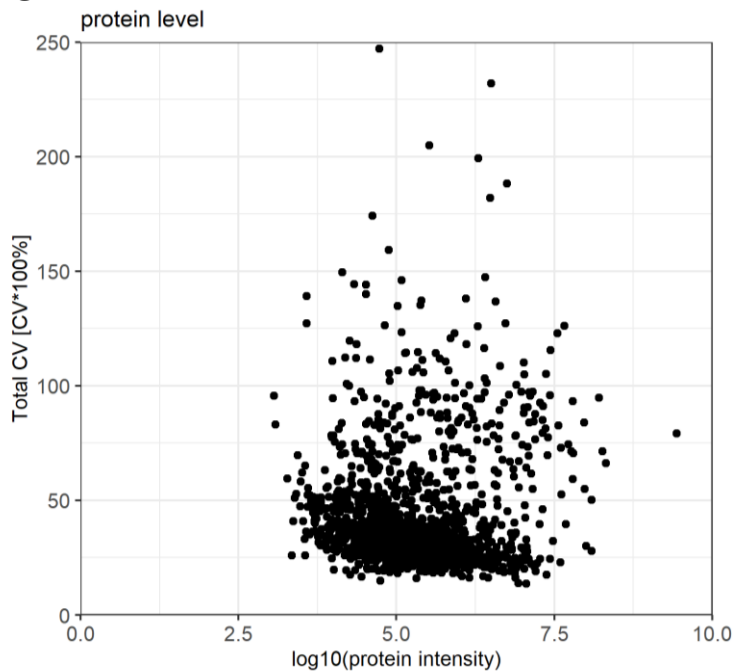**D**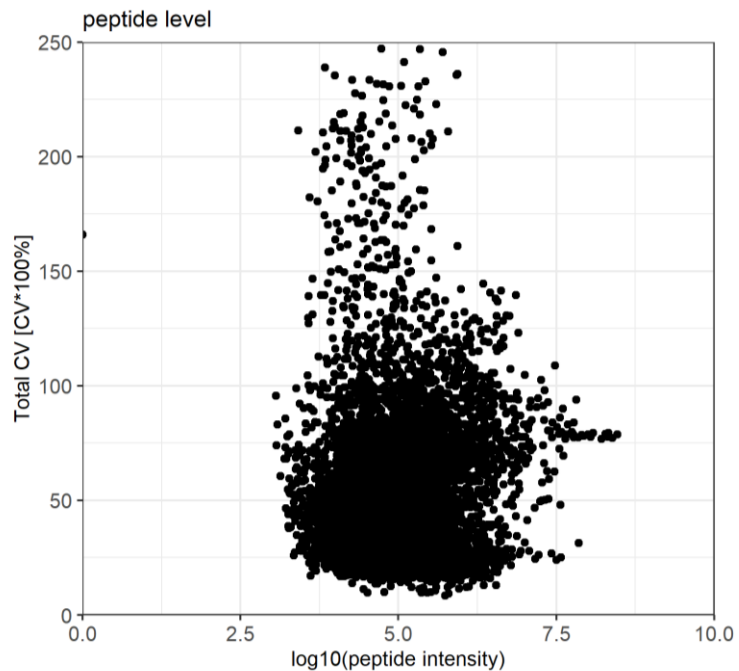

Supplement: Supplementary file 2 — Additional file 2: Figure S1. Dependency of CV on protein and peptide intensities in both pool (set 1) and individual CSF sample sets (set 2). Technical CVs were plotted vs. their corresponding log-transformed protein (A) and peptide intensities (B) in the pool CSF dataset. Likewise, the dependency of total CVs on log-transformed protein (C) and peptide (D) intensities was evaluated for the individual CSF sample dataset. [file 12014_2022_9354_MOESM2_ESM.pdf]

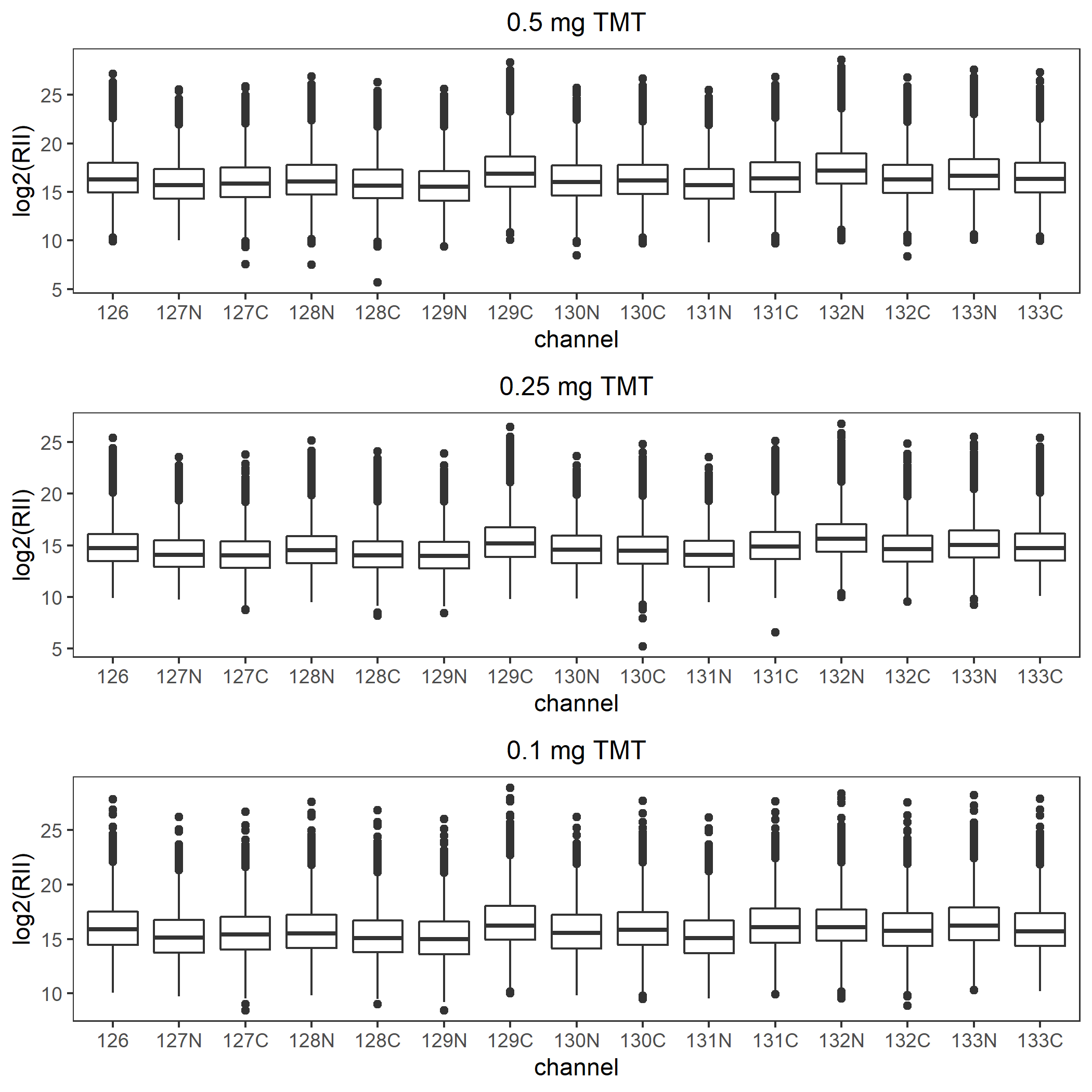

Supplement: Supplementary file 3 — Additional file 3: Figure S2. Reporter ion intensities (RII) per TMT channel in log2-space at differing TMT amounts. For each individual CSF sample corresponding to a specific TMT channel, peptide RIIs were calculated, log2-transformed, and visualized as boxplot. Every sample was labeled with 3 different TMT amounts per 50 µL CSF: 0.5 mg, 0.25 mg, and 0.1 mg. [file 12014_2022_9354_MOESM3_ESM.tiff]

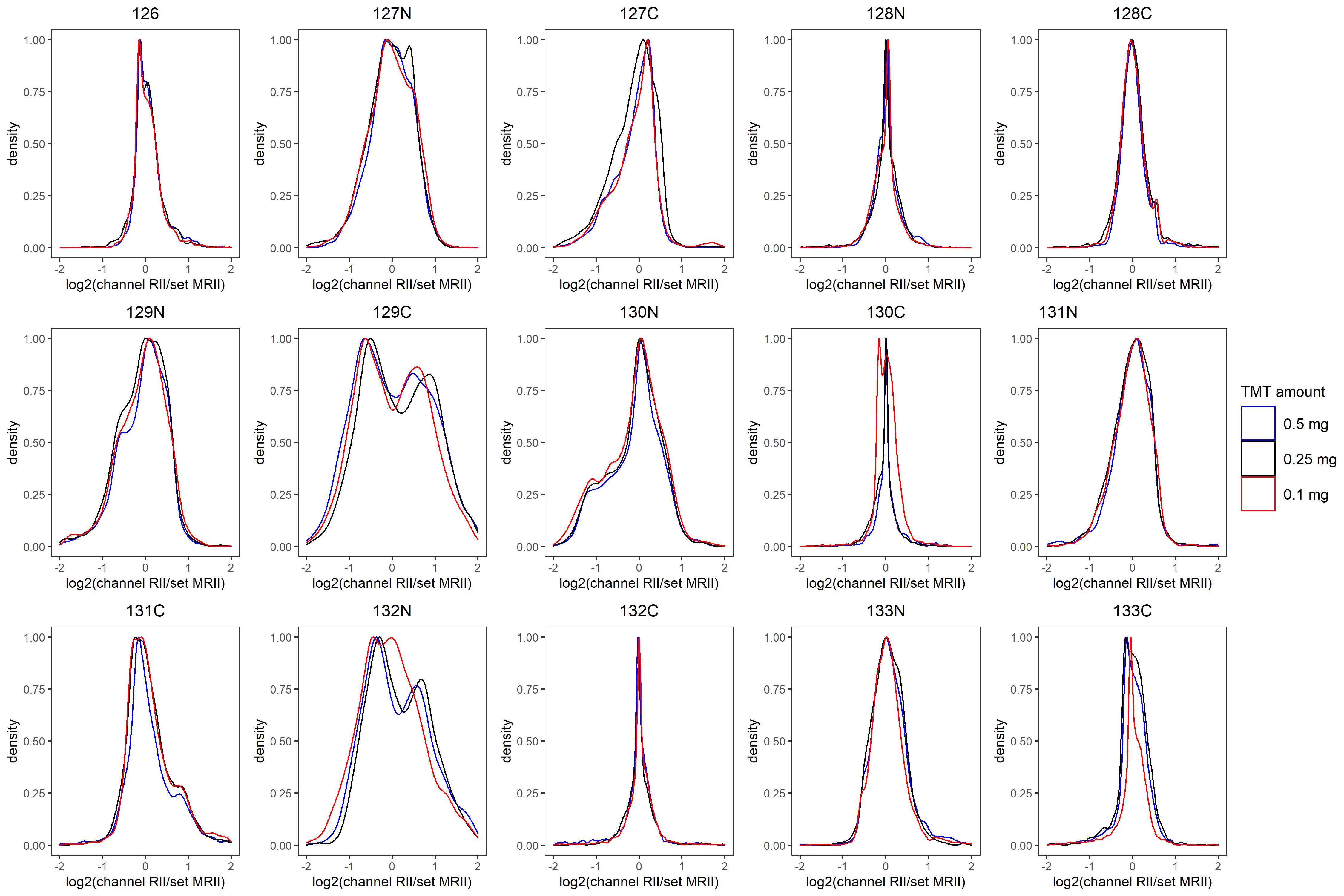

Supplement: Supplementary file 4 — Additional file 4: Figure S3. Distribution of median centred peptide-ratios for each individual CSF sample i.e., channel at differing TMT labeling amounts. Individual CSF samples, corresponding to a specific TMT channel, were prepared separately, and labeled with 0.5 mg (blue), 0.25 mg (black), 0.1 mg (red) TMT per 50 µl CSF. Following LC–MS analysis, the density distribution of log2 peptide ratios (channel reporter ion intensity) was calculated and median centred employing median reporter ion intensity in each sample (MRII). Only peptides common to all three sets were considered for analysis. [file 12014_2022_9354_MOESM4_ESM.tiff]
